# Hypoxia Due to Cardiac Arrest Induces a Time-Dependent Increase in Serum Amyloid β Levels in Humans

**DOI:** 10.1371/journal.pone.0028263

**Published:** 2011-12-14

**Authors:** Henrik Zetterberg, Erik Mörtberg, Linan Song, Lei Chang, Gail K. Provuncher, Purvish P. Patel, Evan Ferrell, David R. Fournier, Cheuk W. Kan, Todd G. Campbell, Ray Meyer, Andrew J. Rivnak, Brian A. Pink, Kaitlin A. Minnehan, Tomasz Piech, David M. Rissin, David C. Duffy, Sten Rubertsson, David H. Wilson, Kaj Blennow

**Affiliations:** 1 Department of Psychiatry and Neurochemistry, The Sahlgrenska Academy at the University of Gothenburg, Mölndal, Sweden; 2 Department of Surgical Sciences, Anaesthesia and Intensive Care, Uppsala University, Uppsala, Sweden; 3 Quanterix Corporation, Cambridge, Massachusetts, United States of America; Nathan Kline Institute and New York University School of Medicine, United States of America

## Abstract

Amyloid β (Aβ) peptides are proteolytic products from amyloid precursor protein (APP) and are thought to play a role in Alzheimer disease (AD) pathogenesis. While much is known about molecular mechanisms underlying cerebral Aβ accumulation in familial AD, less is known about the cause(s) of brain amyloidosis in sporadic disease. Animal and postmortem studies suggest that Aβ secretion can be up-regulated in response to hypoxia. We employed a new technology (Single Molecule Arrays, SiMoA) capable of ultrasensitive protein measurements and developed a novel assay to look for changes in serum Aβ42 concentration in 25 resuscitated patients with severe hypoxia due to cardiac arrest. After a lag period of 10 or more hours, very clear serum Aβ42 elevations were observed in all patients. Elevations ranged from approximately 80% to over 70-fold, with most elevations in the range of 3–10-fold (average approximately 7-fold). The magnitude of the increase correlated with clinical outcome. These data provide the first direct evidence in living humans that ischemia acutely increases Aβ levels in blood. The results point to the possibility that hypoxia may play a role in the amyloidogenic process of AD.

## Introduction

Amyloid β (Aβ) peptides are produced by many cell types in the body but the expression is particularly high in the brain. Accumulation of Aβ in the form of extracellular plaques is a neuropathological hallmark of Alzheimer disease (AD) and thought to play a central role in the neurodegenerative process [1]. In familial AD, brain amyloidosis is caused by mutations in the genes encoding amyloid precursor protein (APP) or γ-secretase; the enzyme complex that together with β-secretase is responsible for producing Aβ from APP [2]. However, very little is known about what drives Aβ accumulation in the brains of patients with sporadic AD.

Mounting evidence suggest that cerebral hypoxia may play a role in the pathogenesis of sporadic AD. Epidemiological data show that cerebrovascular risk factors, such as hypertension, hyperhomocysteinemia and hypercholesterolemia, increase the risk of AD [3], [4], [5], [6]. Mild transient brain hypoperfusion in AD transgenic mice results in an acute increase in Aβ secretion through induced β-secretase (BACE1) protein expression [7], which is regulated by hypoxia inducible factor 1-α (HIF1-α) [8]. In a similar manner, bilateral carotid occlusion in rats results in time-dependent accumulation of oligomeric Aβ in the hippocampus [9]. In humans, a marked increase in Aβ expression, peaking at 4 days post-stroke, is found in pyramidal neurons in the hippocampus of patients who died from ischemic stroke [10]. Finally, a neuropathological study shows that in patients resuscitated after cardiac arrest who died 3–36 days later, APP over-expression is found in cortical and subcortical neurons together with non-fibrillar Aβ plaques in the neuropil [11].

To further elucidate the link between hypoxia and Aβ production in living humans, we employed a new technology (Single Molecule Arrays, SiMoA) capable of ultrasensitive protein measurements [12], [13] to look for changes in serum Aβ42 concentration in serial blood samples from patients following cardiac arrest and resuscitation.

## Methods

### Ethics statement

The study was performed at the general intensive care unit at Uppsala University Hospital, Sweden, and approved by the Human Ethics Committee of Uppsala, Sweden. Written consent for participation was obtained from a legal next of kin, and later from survivors, when considered competent.

### Patients

Twenty-five unconscious patients (8 women, 17 men, age range 25–85 years, mean 62 years) with cardiac arrest were resuscitated with restoration of spontaneous circulation (ROSC). Patients exhibited systolic blood pressure >80 mmHg after ROSC and a Glasgow Coma Scale ≤7. Hypothermia treatment to a body temperature of 32–34°C for 24 hours, ventilation, and pharmacologic support were administered immediately after resuscitation as described [14]. All patients received an arterial line in the radial or femoral artery for blood sampling. Serial blood samples were collected, starting as soon as possible in the emergency phase (within 6 h after cardiac arrest), and continuing at 1, 2, 6, 12, 24, 48, 72, 96, and 108 h after cardiac arrest. Serum aliquots were frozen at −70°C until analysis.

Patient outcome was assessed using the Glasgow-Pittsburgh cerebral performance category (CPC) scale at discharge from the intensive care unit and 6 months later [15]. The CPC scale ranges from 1 to 5, with 1 representing mildest possible neurological deficit (patient is able to return to work), and 4–5 representing the most severe deficit (vegetative) and death. A CPC of 1 or 2 was considered a good outcome and a CPC score of 3–5 a poor outcome, as described [14]. For patients who died after discharge from the ICU, the better of the two scores was used, as recommended by the Utstein templates [16].

### Arrays

Patient serum samples were measured in triplicate by a novel SiMoA Aβ42 assay. The technique involves performing a paramagnetic bead-based ELISA, followed by isolation of individual capture beads in arrays of femtoliter-sized reaction wells [13]. Fifty femtoliter reaction wells were prepared as described [12]. The ends of bundles of 50,000 optical fibers were polished, and one end of each bundle was etched in mild acid solution. Differential etch rates of the optical fiber core and cladding glass of the bundles caused 4.5 µm diameter, 3.5 µm deep wells to be formed, giving arrays of microwells across the bundles. Arrays were mounted in linear groups of eight within glass holders, spaced to correspond with microtiter plate well columns that served as rinse troughs following bead loading.

### Reagents

Paramagnetic carboxy beads (2.7 µm, Varian) were coated with a commercially available monoclonal (Covance 6E10) directed to the N-terminus of Aβ42, and diluted to 5×10^6^ beads/mL in Tris-BSA. Biotinylated detector was a commercially available monoclonal (Invitrogen H31L21) directed to the C-terminus, diluted to 0.1 µg/ml in PBS with newborn calf serum, NCS (PBS/NCS). Reporter streptavidin:β-galactosidase (SβG) was a conjugate of purified streptavidin (Thermo Scientific) and βG (Sigma), diluted to 25 pM in PBS/NCS with 1 mM MgCl_2_.

### ELISA and imaging

Bead-sample incubations and immunocomplex labeling were conducted in conical 96 well plates (Axygen) in three steps, starting with analyte capture, incubation with biotinylated detector, and labeling with SβG. Beads from the ELISA were loaded into microwells by centrifugation at 1,300 g for 10 minutes. Wells containing beads with labeled Aβ42 were visualized by the hydrolysis of enzyme substrate (resorufin β-D-galactopyranoside, RGP, Invitrogen) by βG into fluorescent product. RGP was introduced to the wells during array sealing just prior to imaging. Enzyme-containing wells were imaged by fluorescence microscope fitted with a CCD camera. The images were analyzed to determine the average number of label enzymes/bead (AEB) [13].

### Statistics

Aβ42 levels and changes were compared between outcome groups using unpaired t-tests in GraphPad Prism 5 (GraphPad Software Inc., La Jolla, USA).

## Results

**Figure 1:**
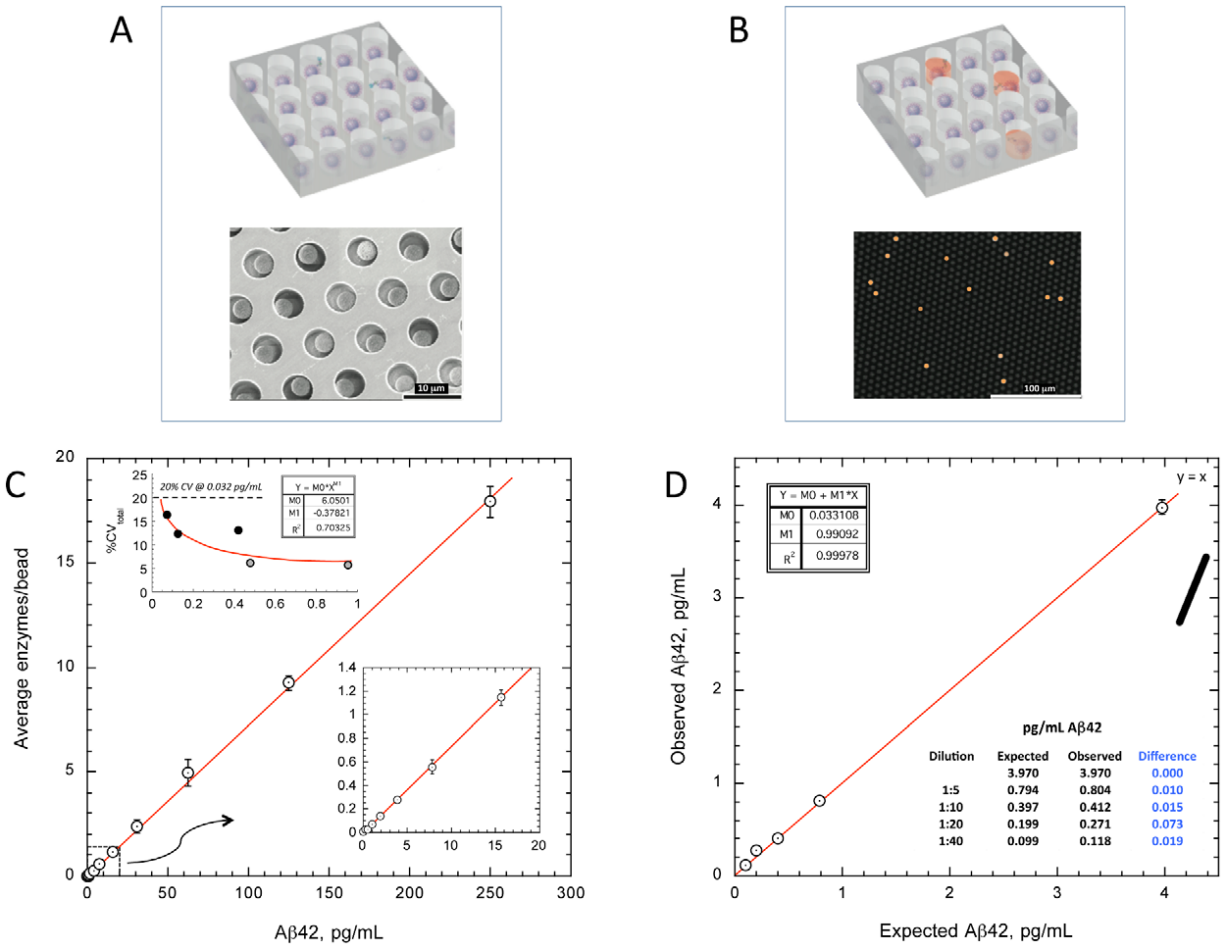
Assay characteristics. Arrays of femtoliter-volume wells permit isolation of 2.7 mm capture beads from a standard bead-based ELISA (**A**), enabling exquisite sensitivity to enzyme label by preventing fluorescent product from diffusing away into a bulk solution. At very low concentrations of Aβ42, beads contain either a single labeled immunocomplex or no complexes, giving rise to digital signal output corresponding to single molecules (**B**). Simultaneous counting of active wells across an array statistically powers estimates of average enzymes/bead. (**C**) Dose-response of digital immunoassay for Aβ42 (n = 3). Y-axis refers to average number of enzyme labels per individual microbead captured in the array. The concentration of label is reduced relative to standard immunoassays, resulting in improved signal∶background at very low Aβ42 concentration. Assay calibrators were purified Aβ42 (Merck) in PBS/BSA. (C inset) Limit of quantification (LoQ) was estimated from total coefficients of variation (CV) from five low panel members (spiked PBS/BSA panels, grey circles, and immunodepleted plasma, black circles) assayed repeatedly across five days. The Aβ42 concentration at which total assay imprecision reached 20% (LoQ) was 0.032 pg/mL. (**D**) Recovery at extremely low Aβ42 concentrations was tested by diluting spiked immunodepleted plasma with PBS/BSA zero calibrator (n = 3). Because samples are pre-diluted 4-fold prior to assay to reduce matrix effects, dilutions include an initial 4-fold dilution.

Singulation of capture beads within microwells permits buildup of fluorescent product from a single enzyme label, making the signal from a single immunocomplex readily detectable (Figure 1A–B). The novel Aβ42 assay has a dynamic range to 250 pg/mL (Figure 1C), with limit of quantification of less than 0.04 pg/mL, enabling precise measurement of low abundance serum Aβ42 in all samples. Precise quantification of peripheral Aβ42 to subpicogram levels has not been previously possible with conventional assay methods. The extreme sensitivity of the assay also permits pre-dilution of the samples, reducing endogenous interferences that have been problematic with assays for this peptide (Figure 1D).

**Figure 2:**
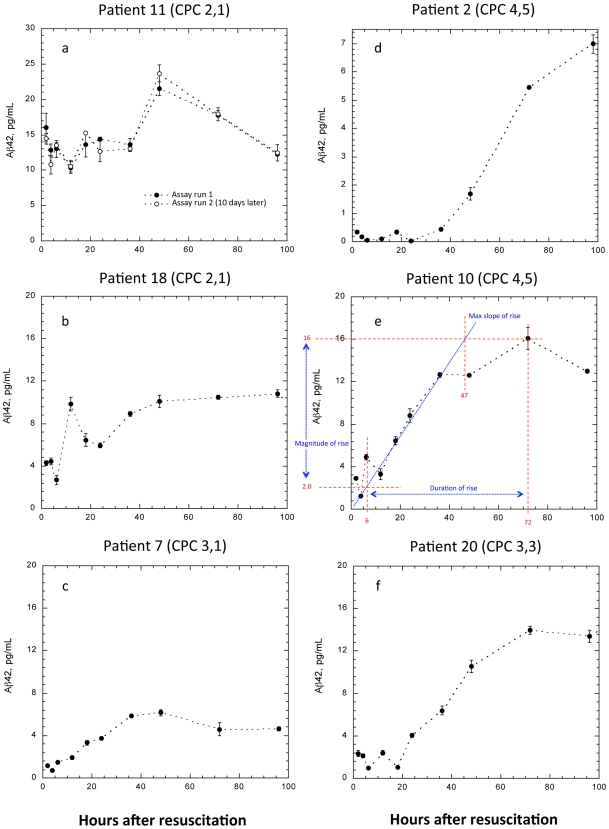
Serum Aβ42 following resuscitation from cardiac arrest. CPC scores depicted are after discharge from the ICU and 6 months later. Panels on left (**A–C**) are profiles from patients exhibiting good outcomes, panels on the right (**D–F**) are from patients with poor outcome. (**E**) Illustration of Aβ42 profile analysis. Baseline Aβ42 was defined as the mean of the two lowest values in the initial 12 hours. The time of initial elevation was defined as the intersection between the baseline Aβ42 and the line of maximum ascension of the major elevation peak. The duration of the Aβ42 increase was defined as the difference between time of initial elevation and time beyond which no significant further rise was observed. The magnitude and maximum slope of rise are also indicated. (**A**) Patient exhibiting smallest relative increase in Aβ42 among all patients. For confirmation, the sample set was re-assayed on a different day. (**D**) Patient with poor outcome exhibiting the largest relative increase from baseline. (**C** and **F**) Patients with similar baseline Aβ42 and poor cerebral outcome upon discharge from the ICU. Six months later, patient BE had recovered good cerebral function, while patient LP had not. Error bars: standard deviation of triplicate measurements.

Figure 2 depicts representative elevation profiles for patients with good and poor 6-month outcomes. Profile parameters were: magnitude of Aβ42 rise, ratio of the peak to baseline Aβ42, slope of Aβ42 rise, and the duration over which the Aβ42 exhibited rising values (Figure 2E). The sum (Aβ42 score) of these parameters was also examined (Table 1). All patients were found to exhibit a significant time-dependent elevation of serum Aβ42. Generally, after a lag period of 10 or more hours, very clear Aβ42 elevations were observed. Elevations ranged from approximately 80% to over 70-fold, with most elevations in the range of 3–10-fold (average approximately 7-fold).

**Table 1:** Characteristics of serum Aβ42 elevation profiles from resuscitated survivors of cardiac arrest during the first 96–108 hours following admission to the intensive care unit.

Patient	Magnitude of rise (pg/mL)		Duration of rise (hr)		Rise ratios (fold increase)		Max slope (pg/mL/hr)		Aβ42 score	
	Good	Poor	Good	Poor	Good	Poor	Good	Poor	Good	Poor
1		17.4				4.10		0.621		
2		6.90		60		70.0		0.150		137
3	11.3		28		4.10		0.315		43.7	
4	11.9		25		4.30		0.466		41.7	
5		4.60		63		5.60		0.242		73.4
6		8.60		78		9.60		0.110		96.3
7	5.07		32		6.45		0.159		43.7	
8		7.10		21		2.45		0.592		31.1
9	9.20		14		6.75		0.657		30.6	
10		14.0		66		8.00		0.340		88.3
11	9.50		15		1.81		0.633		26.9	
12		26.7		18		5.45		1.48		51.6
13	6.50		28		14.0		0.232		48.7	
14	11.0		55		6.50		0.202		72.7	
15	11.0		31		4.90		0.360		47.3	
16		13.3				3.00		0.887		
17	7.20		13		3.13		0.554		23.9	
18	6.90		31		2.97		0.276		41.1	
19		8.80				3.20		0.587		
20		12.3		56		8.70		0.273		77.3
21		9.50		72		16.8		0.132		98.4
22		7.10				31.7		0.372		
23		14.7		33		8.00		0.445		56.1
24		7.90		61		4.30		0.282		73.5
25		7.20		72		15.4		0.141		94.7
Mean	8.96	11.1	27.2	54.6	5.49	13.1	0.385	0.444	42.0	79.8
P value	0.137		0.00091		0.095		0.322		0.00056	

Parameters were sorted on the basis of good or poor 6-month outcome. Blood sampling was terminated early for patients 1, 16, 19 and 22 for medical reasons, and rise durations could not be estimated for these patients. The Aβ42 score equals the sum of magnitude of rise, duration of rise, rise ratio and max slope.

**Figure 3:**
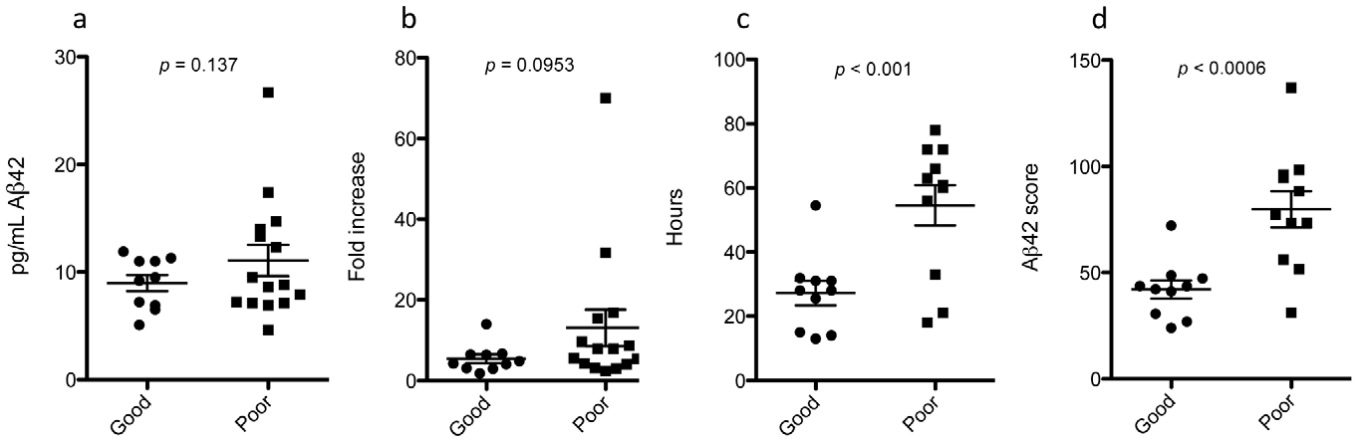
Features of Aβ42 elevation profiles were compared with 6-month overall cerebral outcome. (**A**) Magnitude of Aβ42 rise; (**B**) ratio of maximum to baseline Aβ42; (**C**) duration of major Aβ42 rise; (**D**) sum of **A** to **C** plus maximum slope of Aβ42 rise. Error bars are the standard error of the means.

Aβ42 profile characteristics for each patient are given in Table 1, and graphed in Figure 3. There was no relationship between baseline Aβ42 and outcome (p = 0.53). Large increases in Aβ42, as reflected by rise magnitudes and ratios, exhibited marginal association with poor outcome (student t-test p = 0.137, 0.095 respectively), while a long duration of rise was associated with poor outcome (p<0.001). Slope and total area under the curve (AUC) were not significant (p = 0.32, 0.16 respectively, later not shown). Weak association between AUC and outcome could be due to incomplete expression of the major elevation peak over the sampling interval in many cases (e.g., Figure 2D). Arbitrarily summing the profile parameters further enhanced discrimination (p<0.0006), giving 90% positive and negative predictive values for 6-month outcome using a cut point of 50. Age and gender did not influence the results (p>0.35 for all comparisons).

## Discussion

To our knowledge, these data are the first to link hypoxia with acute elevations in serum Aβ42 levels in living humans. These data corroborate earlier results from animal models, which all suggest a direct relationship between hypoxia and up-regulated amyloidogenic APP-processing [7], [8], [9]. While we make no claim of the observed changes being brain-specific, the data show that circulatory failure with hypoxia dramatically increases Aβ42 levels in blood. Induced Aβ42 production due to hypoxia appears to be the most likely explanation for the results, but increased release from cerebral tissues due to blood-brain barrier breakdown or reduced clearance of the peptide cannot be excluded. The magnitude of the increase is associated with clinical outcome, which in turn probably reflects the severity of the ischemic episode.

A limitation of the study is the lack of a control group. However, earlier results show that the day-to-day variability in Aβ42 levels normally is very low [17]. Moreover, the association of the SiMoA Aβ42 results with clinical outcome suggests a direct relationship between the ischemic episode and Aβ42 levels which is not secondary to the standardized treatment all patients received.

Although there are many differences between acute hypoxia due to cardiac arrest and age-related changes in the cerebrovasculature, it is tempting to speculate that hypoxia induced by arteriolosclerosis and/or lipohyalinosis may play a role in AD pathogenesis by a direct effect on Aβ levels in the brain. On top of this, lowering of pH in hypoxic tissues would further stimulate Aβ oligomerization and aggregation as readily observed in the test tube [18], [19], [20]. A direct effect of hypoxia on Aβ production may provide a putative mechanism for the mounting evidence from epidemiological studies linking cerebrovascular risk factors with AD [3], [4], [5], [6]. It may also help to explain the recently described association of sleep apnea-induced hypoxia with dementia [21].

The data indicate that the SiMoA Aβ42 assay represents a breakthrough in analytical capability for extremely sensitive and reproducible Aβ42 measurement in serum and plasma. The assay allows highly sensitive (limit of quantification 0.032 pg/mL) and precise quantification of Aβ42 in biological fluids. Current immunoassays for measurement of Aβ42 in plasma are based on the ELISA [22], [23], [24] or the Luminex [25] techniques. In addition to lower analytical sensitivity, these immunoassays are hampered by low recovery of spiked Aβ42 into plasma or serum, and as a result the measured concentration of Aβ42 increases when the plasma sample is diluted [25]. These phenomena are due to matrix effects, probably by interaction and binding of Aβ42 to plasma proteins. This means that unrelated plasma proteins contribute to the concentration interpreted to come from Aβ42 in this type of assay. The immunoassay presented here showed highly linear dilution characteristics, together with a recovery of around 100%, suggesting that it gives a very accurate measure of the Aβ42 concentration. This is likely because the interference from plasma proteins is lost when samples are pre-diluted 1∶4 prior to measurement. Because the assay has such high sensitivity, pre-diluting the samples does not compromise the capability of the method to precisely measure low abundance Aβ42. A highly accurate and specific assay for measurement of Aβ42 in blood will be of major importance both for pathogenic studies on AD disease mechanisms, and to monitor an effect on Aβ metabolism of novel therapeutic compounds in clinical trials, a hypothesis that will be tested in future studies.
